# GET WELL: an automated surveillance system for gaining new epidemiological knowledge

**DOI:** 10.1186/1471-2458-11-252

**Published:** 2011-04-21

**Authors:** Anette Hulth, Gustaf Rydevik

**Affiliations:** 1 Swedish Institute for Infectious Disease Control, SE-171 82 Solna, Sweden

## Abstract

**Background:**

The assumption behind the presented work is that the information people search for on the internet reflects the disease status in society. By having access to this source of information, epidemiologists can get a valuable complement to the traditional surveillance and potentially get new and timely epidemiological insights. For this purpose, the Swedish Institute for Infectious Disease Control collaborates with a medical web site in Sweden.

**Methods:**

We built an application consisting of two conceptual parts. One part allows for trends, based on user specified requests, to be extracted from anonymous web query data from a Swedish medical web site. The second conceptual part permits tailored analyses of particular diseases, where more complex statistical methods are applied to the data. To evaluate the epidemiological relevance of the output, we compared Google search data and search data from the medical web site.

**Results:**

In the paper, we give concrete examples of the output from the web query-based system. We also present results from the comparison between data from the search engine Google and search data from the national medical web site.

**Conclusions:**

The application is in regular use at the Swedish Institute for Infectious Disease Control. A system based on web queries is flexible in that it can be adapted to any disease; we get information on other individuals than those who seek medical care; and the data do not suffer from reporting delays. Although Google data are based on a substantially larger search volume, search patterns obtained from the medical web site may still convey more information from an epidemiological perspective. Furthermore we can see advantages with having full access to the raw data.

## Background

During the last couple of years, logs of queries submitted to web-based search engines have received attention as potential sources for infectious disease surveillance. There are several advantages with using web queries for epidemiological analyses:

• We can get information on groups of individuals other than those who see a doctor.

• The data are nearly real-time.

• A system based on web queries can easily be adapted to various diseases.

• The data reflect a point in time close to onset (provided that the person looking for information is actually ill).

This paper describes a fully implemented surveillance system that can generate epidemiological trends from anonymous web query logs. We have named the application GET WELL: Generating Epidemiological Trends from WEb Logs, Like. The system was developed at the Swedish Institute for Infectious Disease Control (SMI). The purpose of GET WELL is to provide a complement to the daily surveillance performed by the epidemiologists at the institute. For example, the generated output can give additional information about a disease, by confirming or contradicting signals of an outbreak from other surveillance systems. In this paper, we present examples of output from GET WELL. We also present a first comparison between data from the general purpose search engine Google and data collected from the medical web site SMI collaborates with, by investigating the amount of epidemiologically useful information that can be obtained from the two sources for two different queries.

In 2006, Eysenbach showed a correlation between clicks on a keyword triggered link on Google and Canadian influenza activity [1]. Queries submitted to the Google search engine, which have shown a correlation with historical influenza data, are used to monitor influenza in a number of countries in an application called *Google flu trends *[2-4]. Influenza queries from another general purpose search engine -- Yahoo -- have also been shown to correlate with official influenza data [5]. Listeriosis is another infectious disease that has been retrospectively analyzed from Yahoo search queries [6]. Statistical models for estimating the influenza burden have been developed on queries submitted to the search engine on a Swedish medical web site [7]. Additionally, a system based on web logs is naturally not restricted to infectious diseases. Cooper et al have studied the correlation between Yahoo queries and common cancer forms in the US [8]. However, infectious diseases are the focus of the presented research as it is to be beneficial for the infectious disease control and prevention in Sweden.

## Methods

### Query data

The search queries, which are the core of the GET WELL application, originate from the search engine on the medical web site Vårdguiden.se [9]. This web site is owned by the Stockholm County Council. Stockholm County covers roughly one fifth of the 9.3 million inhabitants in Sweden. Vårdguiden estimates that 55 per cent of the visitors live in Stockholm County, while the rest live in other parts of the country (or the world) [10]. In November 2009, the web site had 2.5 million visitors (a number which was probably increased due to the influenza pandemic) [11]. All queries submitted to the search engine on Vårdguiden are logged, and date and time are included in each log entry. No information that could potentially reveal the identity of a web site visitor is stored, nor any information that can tie a visitor to a geographical location.

### The application and its implementation

**Figure 1:**
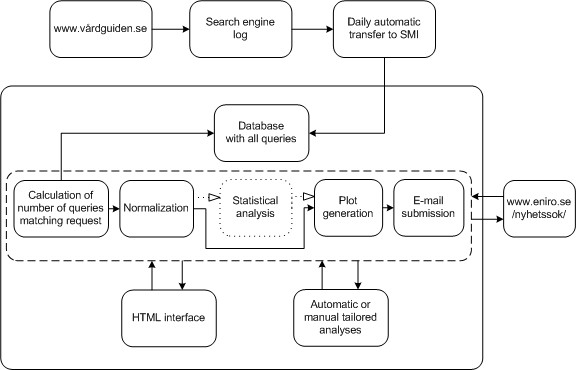
**Flow chart**. A flow chart of GET WELL.

The GET WELL application is available on the SMI intranet since January 2010. In Figure 1, a flow chart of the system is shown. Each night the search log for the previous day is automatically transferred to SMI from the company hosting the Vårdguiden search engine (Euroling). The query terms are extracted from the log and stored in a database, together with the date of submission to Vårdguiden. In the database, we have data from week 27, 2005. The queries are aggregated by week. This aggregation level can easily be altered if required for tailored analyses. For all analyses described in this paper, the results are assumed to reflect the nation as a whole.

The system can be accessed through a designated HTML page on the SMI intranet. On that page, the user can request time series for up to five unique queries for the last five years, counting backwards from the week before the request is made. The matching of a user request to the queries submitted to Vårdguiden is done in a generous fashion. For example, counts on *vomit *will include both *projectile vomiting *and *vomit and pregnancy *(denoted *vomit*, where the star means any prefix or suffix). Also, the matching is done irrespectively of case. When multiple variants of one query type are to be added, the user can specify this by using a pipe sign, for example state *diarre|diarré*, to account for two spelling alternatives of diarrhoea in Swedish. If the search patterns are deemed interesting, more sophisticated analyses of the data can then be initiated. These tailored analyses are initially carried out outside the GET WELL framework, but if successful, may be incorporated into the system.

The result is presented on a web page in the form of a graph. If an email address is provided, the graph is also sent by email. It is also possible to subscribe to emails that are then automatically dispatched by GET WELL. The email can contain either graphs of time series of particular queries or the output from tailored statistical analyses developed in collaboration with the epidemiologist. The emails are normally sent once a week, as this is the default aggregation level of data. Currently there are two diseases (influenza and norovirus infection) for which tailored statistical analyses are made on a regular basis by GET WELL, and for which automatic emails are sent each week to predefined recipients.

It can be assumed that media affect search behaviour, in that more frequently covered subjects will lead to more queries on the subjects in question. For this reason the GET WELL users have an option to also have the number of articles in online news media per week on their subject of choice plotted in the generated graph. This number is fetched from a Swedish search engine for news [12].

The web front-end and the e-mail submission component are implemented in Java. The normalization, the plot generation, and the statistical analyses are implemented in R, while the rest of the components are written in Perl. The query log transfer is realized using Curl. Batch scripts are used to control the flow. A motivation behind the choices of technologies was that they are available as open source software at no cost to the user. Also, Perl is suitable for the required string matching.

### Search volume and normalization

**Figure 2:**
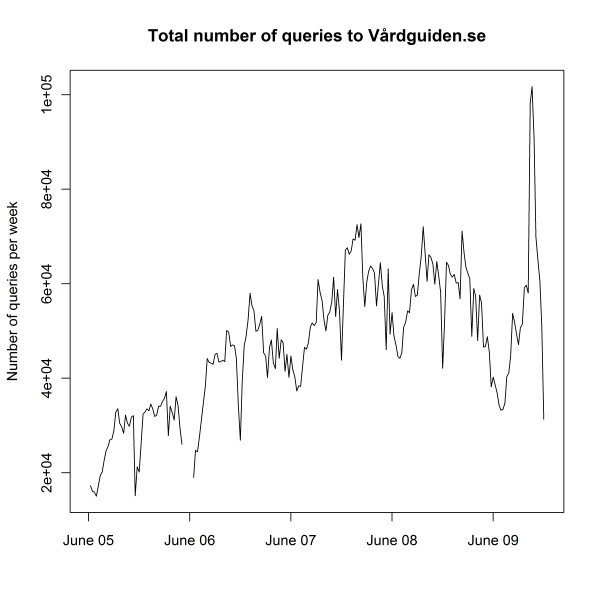
**Total number of queries to Vårdguiden.se**. The total number of queries submitted to the Vårdguiden search engine from June 2005 to December 2009, aggregated by week.

**Figure 3:**
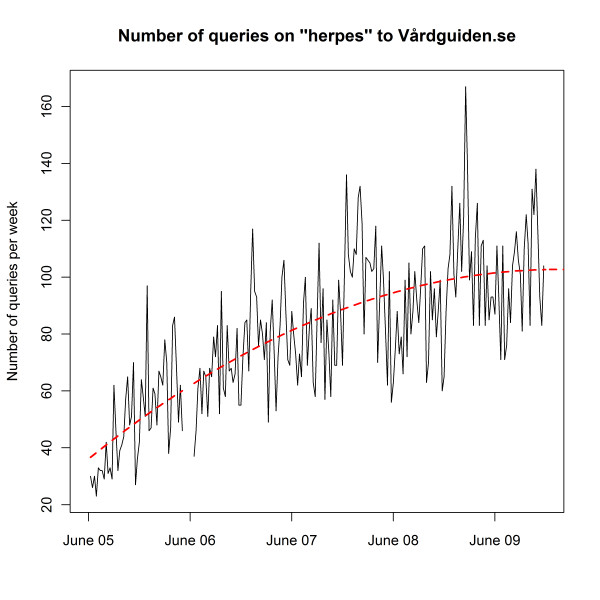
**Number of queries on "herpes" submitted to Vårdguiden.se**. The number of queries on herpes submitted to the Vårdguiden search engine from June 2005 to December 2009, aggregated by week, together with the estimated trend curve (red, dashed line).

In Figure 2, the total number of queries submitted to the search engine per week on Vårdguiden is plotted. In this graph, data from June 2005 to December 2009 are displayed. The broken line in June 2006 is due to five weeks of missing logs. As can be seen in the figure, the total number of queries has increased over time, although not as steeply now as in 2005 and 2006. The large peak in the autumn 2009 was probably due to the influenza A(H1N1) pandemic, as Vårdguiden was one recommended place for information. Because of the increase in search volume over time, we need to normalize each query to get a veracious picture of any divergence from the search trend over time for a particular query. The increase in the total number of submitted web queries per week is large over the period, which means that the difference between January and December is also large. We can therefore not use the total number of queries during a whole year for normalizing. As can be noted in Figure 2, there is also a seasonal variation in the total number of submitted queries, with more queries during the Swedish winter season than during the summer season. This means that we cannot use the total number of queries for a particular week as a denominator for normalization, for we would lose the seasonal variation. For these reasons we investigated a large number of common search terms, to find one without any seasonal variation, which would reflect only the increased usage of the web site. We settled for *herpes*, depicted in Figure 3, a search term which graphically seemed to have little seasonal trend, but a total increase over time that could reflect the increased usage of the web site.

In the current implementation of GET WELL, each submitted query is normalized. This is done by dividing the number of queries by the expected number of searches for that week. The expected number of queries is calculated by using a locally polynomial smoothing function [13] to estimate the increasing trend of queries on herpes. The parameters of the function are chosen in such a way that the resulting curve subjectively captures the non-linear increase. The resulting trend is shown in Figure 3 with a red, dashed line.

## Results

### User requested time series

**Figure 4:**
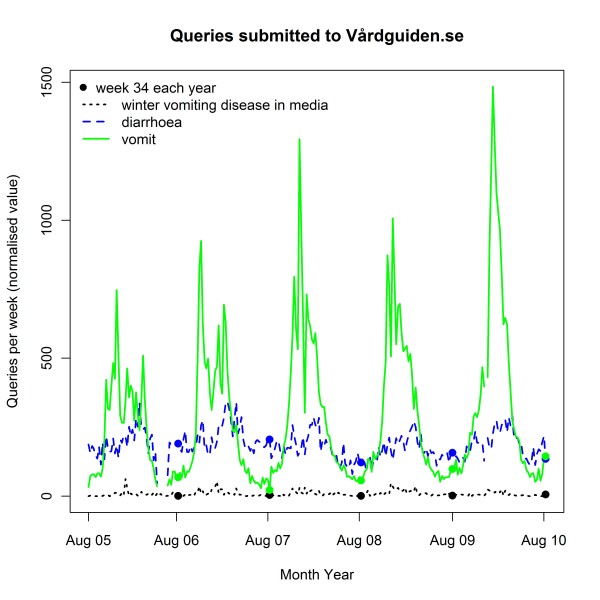
**Queries submitted to Vårdguiden.se**. Example of a graph produced in week 34, 2010 by GET WELL, with media hits. The graph shows the number of articles on winter vomiting disease in Swedish media (black, small dashes), and the normalized number of queries on diarrhoea (blue, larger dashes) and vomit (green continuous) submitted to Vårdguiden, from week 35, 2005 to week 34, 2010. Week 34 each year is marked with a dot.

In Figure 4, we present an example of a time series graph produced by GET WELL for a user entered request. In the figure, time series for the last five years (at the point of request) are shown for two query terms related to the winter vomiting disease: *vomit *(green, continuous line) and *diarrhoea *(blue, large dashes). The graph also shows the number of articles on winter vomiting disease in Swedish online media (black, small dashes). The past week is marked with a dot in the graph for all years, to facilitate comparisons over the years. In the figure, the output from GET WELL was translated to English.

### Search patterns from Vårdguiden versus Google

For the purpose of evaluating analyses on data from a medical web site and from a general purpose search engine, we downloaded data for two selected query terms from *Google Insights for Search *[14]. We extracted the time series for the same two query terms from the Vårdguiden search data. The two terms were the Swedish equivalences to *vomit *and to *influenza*. For both queries we wanted to compare the search patterns obtained from the two web sites. The Google search was restricted to Sweden and the terms in Swedish. The data provided by Google contain the relative search volume, and are already normalized and scaled, while the Vårdguiden data are only normalized. The weeks are differently aggregated: the Vårdguiden data starts the weeks on Mondays, while Google starts the weeks on Sundays.

#### Influenza

The term *influenza *was chosen so that we could compare how the enormous interest in this infectious disease since the beginning of the A(H1N1) pandemic in April 2009 affected the usage of the two sites. It also enabled us to compare the two search engines' search patterns with the traditional influenza surveillance for Sweden. For the Google data, we entered the term *influensa *selecting data for the "last twelve months" (week 50, 2008 to week 49, 2009). For the Vårdguiden data, we counted all queries where *influensa* occurred for the same time period.

**Figure 5:**
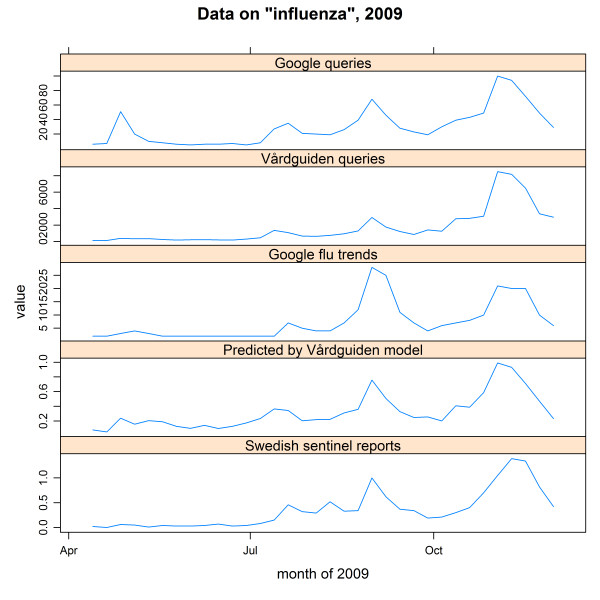
**Data on "influenza", 2009**. From the top: queries on influenza on Google according to Google Insights for Search (data are normalized and scaled); queries on influenza on the medical web site Vårdguiden (data are normalized); Google flu trends estimates; estimates from tailored Vårdguiden model; sentinel influenza data reported to SMI. Data are from week 50, 2008 to week 49, 2009.

Five curves are shown in Figure 5. The two upper curves show data from Google Insights for Search and Vårdguiden on the query *influensa*. To show the benefit of tailored analyses compared to just plotting the time series of a particular query, we have in the graph included the output of Google flu trends for Sweden [3] as well as the output of a tailored influenza model we developed [7], for the same time period. The third curve from the top shows the output from Google flu trends, while the fourth curve shows the output from our tailored influenza surveillance. Both these statistical models were developed on sentinel influenza data from Sweden, and combine a number of different queries related to influenza. In the figure, the "gold standard" of the sentinel reports is also displayed as a reference, showing the proportion of patients with influenza-like illness visiting any of the sentinel general practitioners in Sweden for the same period (the bottom curve).

During the investigated period, influenza received a lot of attention. When visually comparing the results from the two search engines on the query *influensa *in Figure 5, the search patterns are quite similar. The two most pronounced differences are the large peak in May found in the Google data, and the relatively small peak in February for the Vårdguiden data, during the seasonal influenza peak. The Google peak in May reflects the classification of the A(H1N1) epidemic as a pandemic by WHO. The large number of queries on influenza during the A(H1N1) pandemic peak (in November, 2009) hides the height of the seasonal peak in February in the Vårdguiden curve. When visually comparing the output from the two more complex statistical models that incorporate different queries, we can see that Google flu trends have a higher first peak compared to the second peak, while the second peak is higher for the Vårdguiden model. The height of the peaks estimated by the Vårdguiden model is consistent with the sentinel reference data. It is interesting to note that for Google data, the query on influenza alone seems to be a better estimate for the influenza spread in Sweden, with the exception of the huge media attention in April 2009. In summary, the best estimates are produced by the tailored influenza model based on Vårdguiden queries. It is also interesting to note that all four curves based on web queries peak one week before the traditional surveillance.

#### Vomit

The term *vomit *was selected because it is more often part of a compound in Swedish, and might therefore illustrate a potential problem when the user cannot fully control the extraction process. For the Google data, we entered the term *kräkningar *(*kräkningar *is the most common word for the stem *kräk *in the Vårdguiden database). For the Vårdguiden data, we counted all queries where *kräkningar *and *kräk*, respectively, occurred. For the *vomit *analysis we used search queries from week 27, 2005 to week 4, 2010.

**Figure 6:**
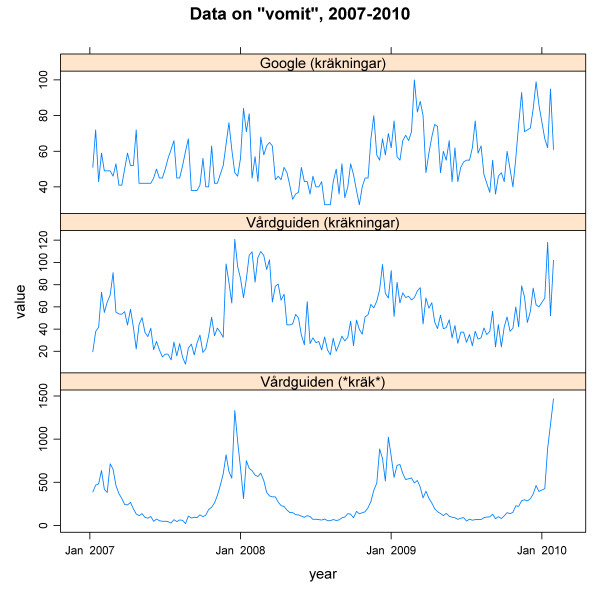
**Data on "vomit", 2007-2010**. Queries on vomit submitted to Google according to Google Insights for Search and to Vårdguiden, from week 1, 2007 to week 4, 2010. The Google data are normalized and scaled. The Vårdguiden data are normalized.

In figure 6, the search pattern for the query *kräkningar *(vomit) obtained from Google Insights for Search is shown, together with two curves from Vårdguiden showing *kräkningar *and *kräk*. In this figure, data from slightly more than three years on the query are displayed (week 1, 2007 to week 4, 2010). We excluded early data (from week 27, 2005) as the Google data contained a large number of zeros for that period. In the Vårdguiden data on *kräkningar*, there is a clearer seasonality compared to the Google data, retrieved on exactly the same query. As previously mentioned, Swedish is rich in compounding and to illustrate how this affects visitors' query formulation, we also plotted the number of queries containing the stem *kräk *(vomit) submitted to Vårdguiden during the same period. As can be seen in the lowest graph in Figure 6, the seasonal pattern of vomiting is then very pronounced. Since the pattern from *kräk* follows the seasonality of the winter vomiting disease, it has a higher epidemic relevance [15].

## Discussion

The traditional surveillance at SMI is mainly based on reports from clinicians and laboratories. GET WELL is developed to serve as a complement to this traditional surveillance. The output from GET WELL can be used to identify epidemiological problems, which then can be investigated further by gathering more detailed information -- such as geographical, age or serotype distribution -- through other sources. GET WELL can also be used to confirm or contradict signals from other surveillance systems, and the time series can be used for regular monitoring. Once relevant search patterns have been identified, evaluations tailored for the disease in question, taking into account the available traditional surveillance are required. The epidemiologists are instrumental in these evaluations. Influenza and norovirus infections are currently monitored on a weekly basis by automatic dispatches from GET WELL. The web query-based surveillance for these two diseases has been evaluated with respect to the traditional surveillance [7,15]. From personal communications, the epidemiologists at SMI in charge of the surveillance of these diseases see great value in the output from the system.

From the comparison between search patterns from Vårdguiden and those obtained from Google Insights for Search, we can see qualitative differences. For example, it seems that substantial media attention affects Google more than it affects the medical web site. Also, an epidemiologically interesting seasonal pattern was found in the Vårdguiden data for the query on vomit, while such a pattern was hardly discerned in the Google data.

It could be that those who search for information out of curiosity rather than illness obscure the epidemiological link between search and disease. One evidence that the specificity is higher in the Vårdguiden data than in the Google data is that the second most common search phrase for vomiting on Google for the investigated period and country is *vomiting dog*, while this query has occurred only five times in total on Vårdguiden during this period. In the context of human epidemiological surveillance, search terms refereeing to animal illness are rarely relevant.

There are several advantages with having access to the raw data. For tailored analyses, we have total control of the data and can transform it, if needed, to the required format. We can also customize the matching process to the investigated language, when counting the number of queries submitted to the web site. As mentioned, Swedish is rich in compounding, and by extracting all queries matching a particular stem, we may get a search pattern which is of greater value in its epidemiological information.

On the other hand, there are other advantages with Google. For example, as Google stores IP addresses, regional information can be provided. (The Vårdguiden query logs are anonymous, which can be a disadvantage from the point of view of surveillance, but from the point of view of the individual who is seeking information on personal health problems, we see it as crucial for the question of personal integrity.) Also, with Google Insights for Search, international comparisons can quickly be made. In addition, data are easily accessible. For a discussion on how Google trends can be used for infectious disease surveillance, see [16,17]. Also, Pelat et al have performed analyses on three diseases on French Google search data [18].

GET WELL is currently only used for monitoring known diagnoses and symptoms. Potentially, web query data could be used as a source for early detection of outbreaks or other health hazards. This type of data is, however, inherently noisy, which means that the number of queries of a particular symptom would need to rise considerably if the system was not to overwhelm a user by false alarms at other times. For the reason of noise, the source is also better suited for diseases with a wider spread. Worth noting though, is the ability of a system like GET WELL to provide reassurance that there are no widespread negative health effects of a particular event. For example, data from GET WELL were used to investigate the development of symptoms that could be caused by ash from the volcanic eruption from Eyjafjallajokull during a couple of weeks in April 2010.

There are a number of parameters to consider when selecting a medical web site for monitoring epidemiological trends. First of all, the representativeness of the visitors, the geographical coverage and the number of visitors should be investigated. Secondly, the accessibility of historical data as well as continuing public availability of the web site need to be ensured. A third parameter -- and in practise the critical one -- is the willingness of those running the web site to have a long-term cooperation. If there are multiple candidate web sites, using several sites could be an option, either by investigating output from different sites independently or by using the data together as input to a single surveillance system. An alternative surveillance source could be the public health departments' own search logs, although initially the representativeness of the visitors must be examined.

## Conclusions

In this paper, we have presented GET WELL, which is a computerized surveillance system based on anonymous logs of queries submitted to the search engine on a Swedish medical web site. The system is now in regular use at the Swedish Institute for Infectious Disease Control. The web queries are a source of information which can be a valuable complement to the traditional surveillance. A system based on web queries is flexible in that it can be adapted to any disease; we get information on other individuals than those who seek medical care; and the data do not suffer from reporting delays.

We also described a comparison between generated time series from the search data on the medical web site (Vårdguiden) with those from a general purpose search engine (Google). Web queries from a medical web site seem to be less affected by curious searchers than a general purpose search engine, and one of the investigated queries showed a substantially more interesting epidemiological pattern. For the other investigated query, the difference in search pattern between the general search engine and the search engine on the medical web site was small. We see, however, that the main advantage with *not *using data from Google is that full access to raw data enables statistical analyses, tailored according to the needs of the epidemiologists. We also strongly feel that public health institutes should have the full responsibility for the public health surveillance. Moreover, collaborating with players in the public sector means that the collaboration is less dependent.

There are limitations of any one source, and epidemiologists should strive at using multiple sources in their analyses. With laboratory data, for example, we suffer from reporting delays, while the sources used for syndromic surveillance usually are pre-diagnostic and thus more uncertain. By examining the output from various systems, a fuller picture of the disease under investigation is given. A system like GET WELL can be one important component in the daily surveillance of infectious diseases in that it can complement the more traditional surveillance, and potentially give rise to new epidemiological insights.

## Competing interests

The authors declare that they have no competing interests.

## Authors' contributions

AH conceived of and implemented the system. GR conceived of and implemented the statistical analyses (except the norovirus surveillance). Both authors contributed to the analyses of the work, and drafted, read and approved the manuscript.

## Pre-publication history

The pre-publication history for this paper can be accessed here:

http://www.biomedcentral.com/1471-2458/11/252/prepub

## References

[B1] Eysenbach G Infodemiology: tracking flu-related searches on the web for syndromic surveillance AMIA Annu Symp Proc 2006 244 8 PMC1839505 17238340

[B2] Ginsberg J Mohebbi MH Patel RS Brammer L Smolinski MS Brilliant L Detecting influenza epidemics using search engine query data Nature 2009 457 1012 4 10.1038/nature07634 19020500

[B3] Google Flu Trends http://www.google.org/flutrends/

[B4] Wilson N Mason K Tobias M Peacey M Huang QS Baker M Interpreting "Google Flu Trends" data for pandemic H1N1 influenza: The New Zealand experience Euro Surveill 2009 14 44 pii = 19386 19941777

[B5] Polgreen PM Chen Y Pennock DM Forrest ND Using Internet searches for influenza surveillance Clin Infect Dis 2008 47 1443 8 10.1086/593098 18954267

[B6] Wilson K Brownstein JS Early detection of disease outbreaks using the Internet CMAJ 2009 180 8 829 31 19364791 10.1503/cmaj.090215 PMC2665960

[B7] Hulth A Rydevik G Linde A Web Queries as a Source for Syndromic Surveillance PLoS ONE 2009 4 2 e4378 10.1371/journal.pone.0004378 19197389 PMC2634970

[B8] Cooper CP Mallon KP Leadbetter S Pollack LA Peipins LA Cancer Internet search activity on a major search engine, United States 2001-2003 J Med Internet Res 2005 7 3 e36 10.2196/jmir.7.3.e36 15998627 PMC1550657

[B9] Vårdguiden http://www.vardguiden.se

[B10] Vårdguiden Labs: Vårdguiden 1 miljon besök http://www.vardguidenlabs.se/2009/02/09/vardguiden-1-miljon-besok/In Swedish

[B11] Vårdguiden Labs: 2,5 miljoner besök i nov http://www.vardguidenlabs.se/2009/12/01/25-miljoner-besok-i-nov/In Swedish

[B12] Eniro nyhetssök http://www.eniro.se/nyhetssok

[B13] R Development Core Team Function "loess" in R 2.9.0 R: A language and environment for statistical computing 2009 R Foundation for Statistical Computing, Vienna, Austria http://www.R-project.org ISBN 3-900051-07-0

[B14] Google Insights for Search http://www.google.com/insights/search

[B15] Hulth A Andersson Y Hedlund KO Andersson M Eye-opening norovirus surveillance Emerg Infect Dis 2010 16 8 10.3201/eid1608.100093 PMC3298324 20678337

[B16] Carneiro HA Mylonakis E Google trends: a web-based tool for real-time surveillance of disease outbreaks Clin Infect Dis 2009 49 10 1557 64 10.1086/630200 19845471

[B17] Brownstein JS Freifeld CC Madoff LC Digital disease detection--harnessing the Web for public health surveillance N Engl J Med 2009 360 21 2153 5 2157 10.1056/NEJMp0900702 19423867 PMC2917042

[B18] Pelat C Turbelin C Bar-Hen A Flahault A Valleron AJ More diseases tracked by using Google trends Emerg Infect Dis 2009 15 8 1327 8 10.3201/eid1508.090299 19751610 PMC2815981

